# Emotion, Psychological Well-Being and Their Influence on Resilience. A Study with Semi-Professional Athletes

**DOI:** 10.3390/ijerph16214192

**Published:** 2019-10-30

**Authors:** Rubén Trigueros, José M. Aguilar-Parra, Joaquín F. Álvarez, Jerónimo J. González-Bernal, Remedios López-Liria

**Affiliations:** 1 Department of Psychology, Hum-878 Research Team, Health Research Centre, University of Almeria, 04120 Almeria, Spain; jalvarez@ual.es; 2 Department of Psychology, University of Burgos, 09001 Burgos, Spain; jejavier@ubu.es; 3 Department of Nursing Science, Physiotherapy and Medicine, Hum-498 Research Team, Health Research Centre, University of Almeria, 04120 Almeria, Spain; rll040@ual.es

**Keywords:** motivation, resilience, anxiety, self-esteem, emotion

## Abstract

The objective of the present study is to analyze the influence of coaches on emotional intelligence and on levels of anxiety, motivation, self-esteem, and resilience among athletes. Five-hundred forty-seven semi-professional athletes between the ages of 16 and 19 participated in this study. Various statistical analyses were conducted which explain the causal relationships between the variables. The results, obtained using a structural equations model, find that while autonomy support positively predicts emotional intelligence, perceived control predicts it negatively. Moreover, emotional intelligence positively predicts self-esteem and self-determined motivation, but negatively predicts anxiety. Other results show that self-esteem positively predicts self-determined motivation, whereas anxiety predicts it negatively. Finally, self-determined motivation positively predicts resilience. Indeed, the study demonstrates the influence and the importance of coaches in relation to the emotional intelligence, psychological well-being, and motivational processes of adolescent athletes when the latter engage in their respective sports. These results help to better understand how different behavioral, emotional, and social aspects belonging to the athlete interrelate with one another during competition.

## 1. Introduction

One of athletes’ main objectives is to develop their own abilities in order to increase their commitment and sports performance [1]. However, it is also necessary for them to consolidate the habits of a healthy lifestyle and fully develop their physical, cognitive, and social capacities [2]. In this sense, coaches can have a significant influence on athletes’ commitment to an activity, primarily because the development of an athlete’s personality and cognitive capacities depends on the degree of interaction maintained by the coach [3]. Thus, on one hand, these individuals must comprehend the importance of each practice as part of a training program in its totality. On the other hand, he/she must also understand and be aware of the transformation and personal changes of each athlete on social, cognitive, and emotional levels [4]. However, to date, studies have mainly examined the coach’s influence on athletes’ motivational processes and their subsequent performance in competition [5], in detriment to their emotional intelligences, which are in fact an important resource for the generation of adaptive behaviors that can help increase athletic performance [6]. Based on the information previously detailed, the objective of the present study is to analyze the influence of coaches on emotional intelligence (EI), as well as on levels of anxiety, motivation, self-esteem, and resilience among athletes. 

Self-determination theory (SDT; [7]) is a macrotheory on human motivation that highlights the influence of interpersonal and social surroundings on human behavior. SDT defends the existence of three different types of motivation (self-motivation, controlled motivation, and demotivation) which greatly influence social relationships, personal well-being, and individual performance. In this regard, self-motivation reflects the reasons why individuals engage in certain behaviors; reasons which are based on a sense of choice, willingness, and ownership toward an action. In contrast, controlled motivation represents a series of behaviors influenced by obligation or pressure from others. Finally, amotivation involves the complete absence of motivation. These last two types of motivation lead to a lack of self-regulation of adaptive behaviors, as people tend to avoid and/or cease an activity due to the absence of rewards, external social recognition, or the use of coercive measures. Conversely, self-motivation facilitates adaptation, as it leads to self-regulation of behavior, mainly because individuals tend to persist due to their own satisfaction produced by the activity [8]. 

According to SDT, the motivation experienced by the individual is influenced by their social context, which is determined by two very different interpersonal styles: Autonomy support or controlling style. Autonomy support refers to the promotion of self-initiative and the mental and physical self-development of the athlete [7]. In contrast, the controlling style is based on external pressures, the use of coercive measures, and obligations. In this case, athletes perceive these strategies as the origin of their behaviors, undermining their own initiative, effort, and personal self-knowledge [9]. 

Various studies examining the field of sports from the perspective of SDT have shown that autonomy support from coaches can contribute to the generation of positive adaptive behaviors among athletes related to, for example, their self-esteem [8], self-concept, and sense of well-being [10]. Conversely, the controlling style employed by a coach is positively associated with high levels of controlled motivation [9], stress, depression [11], and maladaptive behaviors [12]. However, hardly any studies exist which have analyzed the influence a coach has on the athlete’s control over his/her own emotions and emotional state during competition and/or training sessions. Indeed, sports are quite powerful in the sense that they generate a range of emotions during a variety of events which athletes experience while competing or training; not to mention the emotions produced during regular interaction with coaches, teammates, and other players [9,10]. 

Emotional Intelligence (EI) is a relatively new term which is defined as the ability to facilitate the recognition and regulation of emotions that, in turn, facilitate the generation of adaptive behaviors [13]. There are two main theories that seek to comprehend the concept of EI: the trait model [14] and the ability model [15]. The differences between both lie in the fact that trait theory considers EI to be a construct linked to a set of stable personality traits, socio-emotional competences, motivational aspects, and various cognitive abilities which are essential for facing obligations and pressures [14]. In contrast, the ability model views EI as a type of intelligence based on the adaptive use of emotions and their application in our thinking, which allows the individual to adapt to his/her surroundings and solve problems. Despite their differences, both theories share several common elements, such as the fact that emotions are considered predictors of positive adaptive behaviors [16]. 

In recent years, various studies have analyzed the adaptive response of athletes to various adverse events they endure at some point during their athletic life (i.e., extremely demanding physical tasks, competitive failures, encounters with other players, embarrassing situations, and injuries) [17,18]. This response is known as resilience, and it is defined as the set of qualities that represent the athlete’s capacity to overcome unfavorable, unpleasant, and stressful situations. It is also responsible for the positive growth of the individual, as it is involved in the achievement of personal objectives and athletic goals. In short, resilience contributes to the evolution of the exhaustive process of behavioral, social, and emotional adaptation [19]. However, when confronted with an adverse situation, this adaptive response is influenced by the possession and presence of vulnerability factors and protection factors, which are internal and external, and result in a positive adaptation to the risk at hand [20]. 

Anxiety, which is one of the vulnerability factors, is defined as a negative emotional state consisting of a combination of feelings of nervousness, preoccupation, and apprehension related to the activation of the body, which includes a somatic component (physical anxiety) and a cognitive component (mental anxiety) [21]. This level of anxiety activation not only depends on the situation that is causing stress, but also on the individual’s perception of the challenge at hand [22]. 

At the athletic level, and specifically during competition, there are aspects of sports that coincide with certain comparative and evaluative elements, which can easily be identified as adverse and challenging [23]. In this sense, according to García-Mas et al. [24], the phenomenon of state-anxiety associated with the athletic world is that which appears immediately before and during competition. In contrast, trait-anxiety in the athletic world is characterized by a high degree of activation due to enduring successive negative experiences during competition [25]. Unlike state-anxiety, trait-anxiety is not directly manifested in behavior and, therefore, must be assessed according to the frequency with which an individual experience increases in their state of anxiety. Thus, subjects displaying high levels of trait-anxiety perceive a broader range of situations as threatening, and are more prone to suffer from state-anxiety more frequently and intensely. High levels of state-anxiety are perceived as intensely unpleasant; therefore, if an athlete is unable to avoid the stress to which he/she is exposed, that individual will activate the coping mechanisms necessary to face the threatening situation at hand [26]. 

Among the protection factors, the most notable is self-esteem. It is a construct comprised of cognitive and behavioral components, which are characterized by either emotional or physical aspects [27]. The cognitive component relates to the general perception that each individual develops of themselves, and the emotional component refers to the process of self-assessment conducted by the individual [28]. Prior research (e.g., Jawaher et al. [29]) suggests that self-esteem is an important variable in terms of athletic performance, as it is related to psychological well-being and the individual’s own self-confidence, resulting in a consolidated personality which, in turn, will lead to improved physical-athletic performance. Furthermore, self-esteem has been positively linked to self-efficiency [30], achievement and success [31], and participation in sports activity [32].

**Figure 1:**
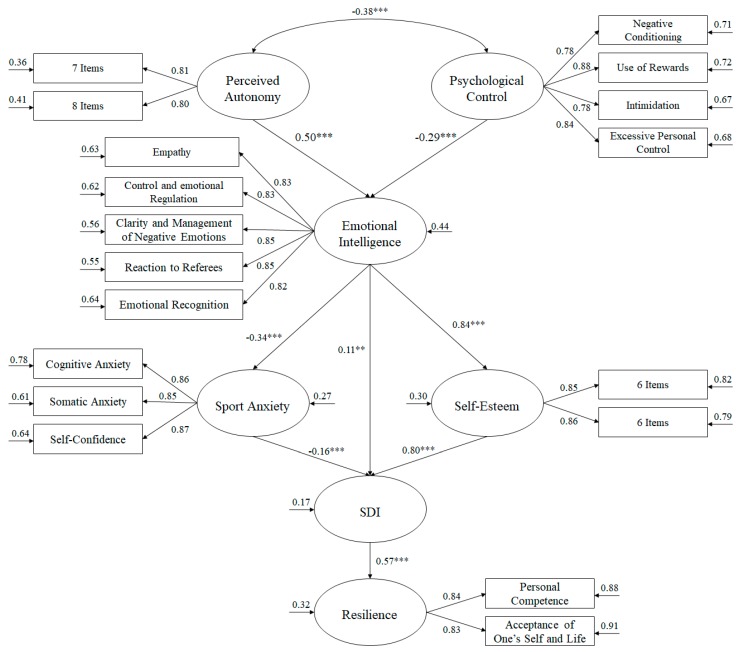
Model of structural equations showing the relationships between the different variables. All parameters are standardized and statistically significant. The variances explained are shown above the small arrows. Note: *** *p* < 0.001; **p < 0.01; * *p* < 0.05. SDI = Self-determination index.

Taking the aspects presented into consideration, the present study seeks to analyze, across structural equation models, the influence of the coach on emotional intelligence and on levels of anxiety, motivation, self-esteem, and resilience among athletes. The purpose of the present study is to analyse the psychological and emotional processes of adolescents while practicing sports. Because adolescents are simultaneously experiencing intense emotions and improving emotional intelligence and emotion regulation, it is a time of great change and potential. The model defends the following hypotheses (see Figure 1): (1) Autonomy support from the coach will positively predict emotional intelligence; (2) use of a controlling style by the coach will negatively predict emotional intelligence; (3) emotional intelligence will positively predict self-motivation and self-esteem, while sports anxiety will be negatively predicted; (4) sports anxiety will negatively predict self-motivation, while self-esteem will be positively predicted; (5) self-motivation will positively predict resilience. 

## 2. Method

### 2.1. Participants

Five-hundred forty-seven semi-professional athletes participated in this study, of whom 289 were male (52.83%) and 258 (47.17%) were female. The participants were between the ages of 16 and 19 (*M* = 17.14; *SD* = 0.81) and belonged to different sports teams from Andalusia (Table 1). To participate in the study, athletes had to provide informed consent signed by parents or legal guardians and be of legal age.

### 2.2. Instruments

Autonomy support. The Spanish version [33] of the Sport Climate Questionnaire, was utilized. This tool was originally derived from the Health Care Climate Questionnaire (HCCQ, [34]), and is comprised of a total of 15 items in its complete version. This survey evaluates athletes’ perception of the degree of autonomy support given by their coaches. Each item begins with the phrase: “In my sport…” and the answers are registered on a 7-item Likert scale, which ranges from not true at all (1) to very true (7).

Controlling coaching style. This aspect was assessed using the Spanish version [35] of the Controlling Coach Behaviors Scale (CCBS, [9]), comprised of 15 items divided into four subscales (controlling use of rewards, negative conditional attention, intimidation, excessive personal control). Each item begins with the phrase: “On my (name of sport) team…” and responses are registered on a Likert scale which ranges from totally disagree (1) to totally agree (7). 

Emotional Intelligence. This aspect was evaluated using the emotional intelligence scale, which was validated for the Spanish context by Arruza et al. [36]. The scale is comprised of a total of 31 items divided among the five factors that compose the scale. Of the five factors, seven correspond to empathy, seven items correspond to control and emotional regulation, four items correspond to clarity and management of negative emotions, three items correspond to referee reactivity, and eight items correspond to emotional recognition. The answers are registered on a Likert scale from totally disagree (1) to totally agree (5). 

Self-Esteem. The tool used in this case was the Spanish version (Balaguer et al. [33]) of the Self-Worth Subscale of the Self-Description Questionnaire (SDQ-III; [37]). It is composed of 12 items and asks athletes to indicate their level of agreement with statements related to how they perceive themselves. The answers are registered on a Likert scale from totally false (1) to totally true (6). 

Anxiety. This aspect was evaluated using the Spanish version of the Revised Competitive State Anxiety Inventory-2 (CSAI-2R) by Cox, Martens, and Russell [38], and validated and adapted by Andrade, Lois, and Arce [39]. This instrument is comprised of 16 items distributed among three factors, of which six correspond to cognitive anxiety, five correspond to somatic anxiety, and five correspond to self-confidence. The answers are expressed according to a Likert scale on which 1 corresponds to not at all, and 4 corresponds to very much so.

Self-Determined Motivation. The instrument used in this evaluation was the Behavioral Regulation in Sport Questionnaire (BRSQ; [40]), which was validated and adapted for the Spanish context by Viladrich, Torregrosa, and Cruz [41]. This scale is comprised of 24 items divided evenly among six subscales utilized for evaluating reasons for participating in sports. These subscales include intrinsic motivation, integrated regulation, identified regulation, introjected regulation, external regulation, and demotivation. All the items share the initial phrase “I participate in this sport…”, followed by differential content according to the subscale. The items are assessed from 1 (completely false) to 7 (completely true). 

The evaluation of Self-Determined Motivation was performed using the self-determination index (SDI; [42]), which was calculated based on the following formula: 3 × intrinsic motivation, 2 × integrated regulation, 1 × identified regulation, −1 × introjected regulation, −2 × external regulation, and −3 × demotivation. Various works have shown this index to be valid and reliable, and it is applied to obtain a value which makes possible to quantify the level of self-determination. 

Resilience was evaluated with the Resilience Scale in the Sports Context (RSSC, [43]), adapted from the Portuguese version developed by Vigário, Serpa, and Rosado [44]. This questionnaire begins with the heading, “Based on your athletic experiences, indicate your level of agreement or disagreement with the following sentences”. The scale is comprised of 25 items distributed between two factors. Seventeen items correspond to personal competence and eight correspond to self-acceptance and sport-life. Respondents indicate their answers according to a Likert scale from 1 (disagree) to 7 (totally disagree). 

### 2.3. Procedure

In order to conduct this study, permission was initially requested from the University of Almeria’s Bioethics Committee for Human Research (Ref. UALBIO 2019/014) to contact various sports clubs throughout Andalusia, for the purpose of subsequently requesting consent to conduct surveys among their young athletes. Prior to beginning the survey process, the clubs and athletes were informed of the study’s objectives. Given that most of the athletes were underage, their parents or guardians were asked to fill out and sign an authorization document before the scales were applied. The questionnaire was administered under the supervision of a professional surveyor from the research group who explained information and answered any questions that arose during the process. The estimated time to respond to the questionnaire was about 25 min. 

### 2.4. Data Analysis

Firstly, the descriptive statistics were calculated. Then, using the Pearson correlation, a correlation analysis was performed between the study variables with the program SPSS v25 (IBM, Armonk, NY, USA). Subsequently, the hypothesized prediction model was tested utilizing the Structural Equations Model (SEM) in the statistics program AMOS v19 (IBM, Armonk, NY, USA). 

In order to apply the SEM, maximum likelihood estimation was utilized, along with a bootstrapping procedure. The tested model was assessed using various fit indices: *χ^2^/df*, CFI (Comparative Fit Index), IFI (Incremental Fit Index), RMSEA (Root Mean Square Error of Approximation) plus its confidence interval (CI) at 90%, and SRMR (Standardized Root Mean Square Residual). Values for *χ*^2^/*df* less than 3, values for incremental indices (CFI, IFI) close to or greater than 0.95, and values for RMSEA and SRMR less than or very close to 0.06 and 0.08 were considered, respectively, as indicating suitable model fit to the data [45]. However, Marsh, Hau, and Wen [46] state that these cut-off values should be interpreted cautiously, as they prove too restrictive and difficult to obtain when testing complex models. 

## 3. Results

### 3.1. Preliminary Results

Table 2 displays the descriptive statistics and existing correlations between the variables studied. As can be seen, the reliability analysis conducted using Cronbach’s alpha obtained a value higher than 0.70 for each of the study variables, whose values were 0.85 for perceived psychological control, 0.91 for perceived autonomy support, 0.87 for emotional intelligence, 0.91 for sports anxiety, 0.93 for self-esteem, and 0.84 for resilience. 

As for the Pearson correlation analysis, it can be observed that psychological control correlated negatively to autonomy support, emotional intelligence, self-esteem, SDI, and resilience, and positively to sports anxiety. The same analysis determined that autonomy support correlated positively to emotional intelligence, self-esteem, SDI, and resilience, and negatively to sports anxiety. Emotional intelligence correlated positively to SDI, self-esteem, and resilience, and negatively to sports anxiety. It was also found that SDI correlated positively to self-esteem and resilience, and negatively to sports anxiety. Self-esteem correlated positively to resilience and negatively to sports anxiety. Finally, resilience correlated negatively to sports anxiety. 

### 3.2. Structural Equations Model Analysis

Prior to testing the hypothesis model using an SEM and analyzing the existing relationships between the variables belonging to the model, the number of existing variables was reduced, as each one had at least two indicators due to the complexity of the model [47]. More specifically, the existing variables used were: Controlling coaching style, which included four indicators (intimidation, excessive personal control, use of rewards, and negative conditioning) [35]; emotional intelligence, which included five factors (empathy, control and emotional regulation, clarity and management of negative emotions, reactions to referees arbitral and emotional recognition) [36]; anxiety, which included three factors (cognitive anxiety, somatic anxiety, and self-confidence) [39]; resilience, which included two factors (personal competence and acceptance of one’s self and sport-life) [43]; and, finally, autonomy support, whose 15 scale items had to be separated into two indicators, as was the case with the 12 items of self-esteem. This procedure was followed to be able to design the model, precisely as suggested by McDonald and Ho [47]. 

The model for the hypothesized predictive relationships (Figure 1) revealed the following fit indices: *χ*^2^ (144. *N* = 547) = 478.72, *p* < 0.001; *χ*^2^/*df* = 3.32; CFI = 0.96; IFI = 0.97; RMSEA = 0.054 (IC 90% = 0.052–0.061); SMR = 0.038.

The relationships obtained between the different factors comprising the model are described as follows:

(a) The correlation between psychological control and autonomy support were negative (β = −0.38, *p* < 0.001).
(b) Psychological control negatively predicted emotional intelligence (β = −0.29, *p* < 0.001). Autonomy support positively predicted emotional intelligence (β = 0.50, *p* < 0.001).
(c) Emotional intelligence positively predicted SDI (β = 0.62, *p* < 0.05), negatively predicted anxiety (β = −0.34, *p* < 0.001), and, finally, positively predicted self-esteem (β = 0.83, *p* < 0.01).
(d) Sports anxiety negatively predicted SDI (β = −0.12, *p* < 0.001) and self-esteem positively predicted SDI (β = 0.74, *p* < 0.001).
(e) SDI positively predicted resilience (β = 0.57, *p* < 0.001).

## 4. Discussion

The aim of the present study was to analyze the influence of the coach on emotional intelligence and on levels of anxiety, motivation, self-esteem, and resilience among young athletes. Until now, studies have mainly examined the influence of the coach on motivation processes among athletes and this group’s performance in competition [5], based on the understanding that emotions have substantial impact on the generation of adaptive behaviors, such as resilience, which can increase athletes’ performance [19]. In this regard, the present work contemplates the study of the athlete’s emotional intelligence, as the ability to recognize emotions and control them can lead to improved performance during competition [48]. Moreover, resilience is also regarded as a determining factor which contributes to a higher level of adaptation on behavioral, emotional, and social levels during competition [49]. 

Various studies in the field of sports have confirmed the positive effect of autonomy support in relation to positive emotions [50] and the negative effect that the controlling coaching style has on positive emotions [51]. However, there is hardly any evidence of research that has addressed the influence of the coach on athletes’ EI, despite the fact that coaching style can have either a counterproductive or favorable effect on how young athletes adopt strategies towards coping with emotions and understanding those of others [52]. The results of the present study have shown that autonomy support positively predicts athletes’ EI, while it is negatively predicted by controlling conduct (Hypothesis 1 and 2). As previously stated, few studies in the field of sports have analyzed the influence of the coach on athletes’ EI. However, a study conducted on emotional labor by Johnson and Spector [53] verified that autonomy support given by the superior acted as a predictor of employees’ emotional intelligence and negatively predicted this group’s negative emotions. Similarly, Wu [54] showed that autonomy support acted as a negative predictor of stress, and a positive predictor of employees’ emotional intelligence and performance in the workplace. Despite the existence of these studies, there have been none in the field of sports or any other that have analyzed the influence of the controlling style on emotional intelligence. Thus, the results of the present study prove to be in line with the findings of those previously mentioned, and with the postulates of SDT. In this sense, the autonomy support offered by coaches fosters athletes’ self-initiative, mental self-development, and personal self-discovery, which in turn favors the development of EI [55]. In contrast, and following the postulates of SDT, it can be inferred that the controlling behavior of a coach will lead to feelings of inability and rejection among athletes, which will diminish their capacity to recognize emotions, which will further undermine their EI. 

The results also demonstrated that EI positively predicted self-esteem and autonomy support, and negatively predicted athletes’ anxiety (Hypothesis 3). These results are similar to those of various studies related to self-esteem in contexts different from the field of sports. According to a study conducted by Rey, Extremera, and Pena [56] with adolescents, EI was positively associated with self-esteem and life-satisfaction. Similarly, a study carried out by Aouani et al. [57] found that EI was positively related to self-esteem and perceived social support, whereby both factors had a significant effect on life-satisfaction. In the context similar to sports, namely physical activity, a study conducted by Bhochhibhoya, Branscum, Taylor, and Hofford [58] showed that those adolescents who engaged in physical activity experienced favorable effects on the development of their emotional intelligence, which consequently resulted in an improved physical self-concept and personal self-esteem. 

With regard to self-motivation, the results of the present study are similar to those obtained in the study by Kajbafnezhad et al. [59] with elite athletes. In this case, the authors analyzed differences related to motivation, emotional intelligence, and psychological state among those who participated in team sports and individual sports. Their study revealed a difference between both groups with respect to motivation and psychological state, but not in relation to EI. However, despite the differences between both groups, the study showed that EI was positively related to self-motivation and psychological well-being. As for a review by Laborde, Dosseville, and Allen [60], these authors also demonstrated a positive effect of EI on athletes’ self-motivation. Finally, regarding sports anxiety, different studies in the field of sports have obtained similar results to those of the present study. One such study conducted by Lu, Li, Hsu, and Williams [61] demonstrated that athletes’ emotional intelligence was negatively related to anxiety, as recognition of this emotion implies the subsequent dominance of said emotion. In addition, a study by Laborde et al. [62] with athletes in active competition determined that high levels of emotional intelligence favored emotional regulation, thereby managing negative emotions and increasing performance during competition. 

With regard to other findings, this study found that self-esteem positively related to self-motivation, while sports anxiety showed a negative relationship with self-motivation (Hypothesis 4). These results are in line with numerous studies in the field of sports in which, for example, self-esteem proved to be a predictor of self-motivation [27,63] and sports anxiety was a negative predictor of self-motivation [24,26]. In this sense, when the athlete displays high levels of self-esteem, he/she assimilates better sports content, concepts, and experiences, thereby developing positive attitudes towards participating in sports, which in turn leads to an athlete’s internal motivation and improved athletic performance. In contrast, when an athlete possesses high levels of anxiety, this state leads to a series of maladaptive behaviors (e.g., stress, inability to concentrate, and mental block) due to fear of losing, of the importance of the event itself, or the uncertainty of a possible injury. Such fears cause the athlete to experience controlled motivation, or even demotivation, as elements external to the athlete are identified as the cause of his/her success or failure. 

Finally, the results of this study also showed that self-determined motivation positively predicted resilience (Hypothesis 5). However, as highlighted by Putnick, Hahn, Hendricks, and Bornstein [64], no empirical evidence can be found in the literature which relates self-motivation to resilience, despite the existence of studies that link self-motivation to personal well-being, on both emotional and cognitive levels [65]. This connection seems logical, as athletes who display self-motivation will be able to cope with various adverse and potentially stressful circumstances they may encounter over the course of their athletic career, thanks to high and significant levels of effort, consistency, dedication, and sacrifice. 

In this way, the relationships between trainer and athlete are complex. This study seeks to raise awareness among trainers of the impact of their interpersonal styles on young athletes’ emotional intelligence, motivation, and psychological well-being. Thus, coaches should not focus exclusively on the athlete’s technical, strategic, and tactical skills, but should also focus on developing effective relationships with their sportspeople.

Indeed, the results obtained in the present study support the postulates of SDT with the introduction of new variables and show its applicability in the Spanish context. The model appears to display good robustness and capacity for generalization, and, to some extent, it also helps to better understand the role of the coach with respect to self-esteem, anxiety, and sports motivation. Nevertheless, with regard to the existing findings using this model, it is necessary to reiterate that this approach is a correlational study, meaning it does not allow cause-effect relationships to be extrapolated, and the results obtained could be interpreted in multiple ways depending on the perspective of the individual. Thus, the present study sought to present possibilities rather than causality, so as to be able to explain the existing relationships between the variables of the study. Continuing in this line, future studies should analyze in depth the results obtained using a longitudinal study which could identify the evolution of the relationship between the coach and the athlete over the course of years working together. Furthermore, it would be interesting to know the influence of motivation, emotional intelligence, and emotion regulation, based on the fact that the way they are perceived varies as adolescents grow, their life experience increases, and they begin to make their own decisions. 

## 5. Conclusions

These results may be of interest to athletes because they suggest that support for autonomy positively predicts emotional intelligence, while psychological control negatively predicts it. As for emotional intelligence, it positively predicts self-esteem, autonomous motivation, and negatively predicts sports anxiety. In addition, self-esteem positively predicts autonomous motivation, while it is negatively predicted by sports anxiety. Finally, autonomous motivation positively predicts resilience. This study successfully shows the importance of coaches being attentive to the emotions of athletes as they model appropriate emotion regulation and promote resilience among adolescents.

## Figures and Tables

**Table 1:** Sociodemographic characteristics.

Participants	*N*
Male	289
Volleyball	76
Handball	84
Basketball	129
Female	258
Volleyball	77
Handball	78
Basketball	103

**Table 2:** Descriptive statistics and correlation between variables.

Variables	M	SD	1	2	3	4	5	6	7
1. Perceived autonomy support	4.44	1.30		−0.52 ***	0.57 ***	−0.29 ***	0.56 ***	0.57 ***	0.36 ***
2. Psychological control	1.81	1.02			−0.50 ***	0.39 ***	−0.44 ***	−0.44 ***	−0.27 ***
3. Emotional intelligence	3.94	1.46				−0.34 ***	0.77 ***	0.77 ***	0.53 ***
4. Sports anxiety	1.87	1.26					−0.39 ***	−0.34 ***	−0.20 ***
*5.* SDI	11.58	12.43						0.81 ***	0.55 ***
6. Self-esteem	4.88	1.71							0.53 ***
7. Resilience	4.02	1.11							

*** *p* < 0.01. Note: M = Mean; SD = Standard Deviation; SDI = Self-Determination Index.
